# How should hospital reimbursement be refined to support concentration of complex care services?

**DOI:** 10.1002/hec.3525

**Published:** 2017-05-19

**Authors:** Chris Bojke, Katja Grašič, Andrew Street

**Affiliations:** ^1^ Centre for Health Economics University of York York United Kingdom

**Keywords:** complex care services, diagnosis related groups, healthcare resource groups, hospital payment

## Abstract

The English National Health Service is promoting concentration of the treatment of patients with relatively rare and complex conditions into a limited number of specialist centres. If these patients are more costly to treat, the prospective payment system based on Healthcare Resource Groups (HRGs) may need refinement because these centres will be financially disadvantaged. To assess the funding implications of this concentration policy, we estimate the cost differentials associated with caring for patients that receive complex care and examine the extent to which complex care services are concentrated across hospitals and HRGs. We estimate random effects models using patient‐level activity and cost data for all patients admitted to English hospitals during the 2013/14 financial year and construct measures of the concentration of complex services. Payments for complex care services need to be adjusted if they have large cost differentials and if provision is concentrated within a few hospitals. Payments can be adjusted either by refining HRGs or making top‐up payments to HRG prices. HRG refinement is preferred to top‐payments the greater the concentration of services among HRGs.

## 1 INTRODUCTION

Evidence indicates that outcomes following treatment are superior in places that perform more of the treatment in question (Bachmann et al., 2003; Hillner, Smith, & Desch, 2000; Skipworth et al., 2010; Smith, 2002). Although a general causal link between the volume of activity and outcomes has not been established definitively (Harrison, 2012), England is moving toward concentrating the provision of some types of service in specialist centres rather than having them delivered in general hospitals (NHS England, 2014a). For instance, from 2011 onwards, stroke patients in London have been admitted into one of eight hyperacute stroke units, providing more complex care to stroke patients, which led to improved overall outcomes (Morris et al., 2014). Concentration is particularly important for patients with relatively rare and complex care needs. Delivery of services for such patients requires a skilled team of staff, and concentrating services in dedicated units is deemed the only way to ensure that volumes are sufficient to ensure best possible outcomes (NHS Specialised Services, 2010).

Such concentration of services means that these skilled teams will treat patients that differ systematically from those treated in general hospitals, differences that may impact the cost of treatment. If the reimbursement system does not account for such differences, hospitals that treat more costly patients will be financially disadvantaged, at the risk of undermining the policy toward greater concentration.

In many countries, hospitals are reimbursed according to the amount and type of activity that they perform, with the type of activity described using some form of Diagnosis‐Related Groups (DRGs; Busse et al., 2013). The English classification is known as Healthcare Resource Groups (HRGs) and, as with all DRG systems, is based on the underlying principle that the constituent HRGs are both clinically meaningful and resource homogenous (Grašič, Mason, & Street, 2015).

Resource homogeneity provides the rationale for reimbursing hospitals using HRGs under a prospective payment system, with the HRG prices usually based on average costs as reported by hospitals that provide that service (O'Reilly et al., 2012). This payment arrangement works well if variation in costs within HRGs is either not related to observable patient characteristics or, if there is a relationship, patients are randomly distributed across hospitals. But if systematic variation in costs is associated with particular groups of patients and hospitals, problems arise: the payment system may financially penalize hospitals treating higher cost patients or simply deter hospitals from treating patients expected to have higher costs (Dranove, 1987).

Systematic variation will arise if treatment costs for patients who receive complex care services are higher than for other patients allocated to the same HRG. We assess this possibility by calculating the cost differential associated with providing complex care services. Hospitals will face greater adverse financial consequences the greater the differential and the higher the proportion of their patients who require complex care. To establish which hospitals are at risk of financial disadvantage, we construct Gini coefficients to summarise the concentration of each type of complex care service among hospitals (Daidone & D'Amico, 2009).

If there is evidence of systematic variation, there are two approaches to adjusting payments in order to mitigate adverse financial consequences. The first is to recalculate the HRG price, so that a top‐up payment is paid for those that receive complex care with an offsetting price reduction for all other patients allocated to the same HRG. The price differential would reflect the estimated cost differential for each particular type of complex care. This first option is easiest to implement because it requires no change to the underlying architecture of the patient classification system.

The second option is to refine the underlying HRGs to which patients are allocated. Hafsteinsdottir and Siciliani set out the theoretical implications of refinement in relation to two treatments, demonstrating that refinement is optimal if otherwise there is a risk of under‐provision of the more costly treatment (Hafsteinsdottir & Siciliani, 2010). We apply this reasoning to the practical challenge of having to consider not just two but the hundreds of different treatments categorised by DRG or HRG systems (Busse et al., 2013).

Refinement of HRGs is most appropriate when patients that receive complex care are concentrated in a small number of existing HRGs. If patients are spread across many HRGs, subdividing them will generate many more HRGs, containing fewer patients. If HRG refinement is coupled with a policy of concentrating complex services in fewer hospitals, there is a risk that a single hospital provides all the care to patients in a particular HRG. In such circumstances, the price for that HRG will be determined by just one hospital, with prospective payment collapsing to cost‐based reimbursement. The option of refining HRGs, then, becomes less attractive the more HRGs that complex care patients are spread across. To assess how patients are distributed across HRGs, we calculate concentration ratios to show the concentration of each type of complex care activity among HRGs (Siegfried, 1975).

Whether or not to refine HRGs requires making decisions about what size of cost differential is deemed material and about how concentrated services need to be among hospitals and HRGs before refinement is considered. These are essentially policy decisions but, for illustrative purposes, we show which complex care services would be candidates for either top‐up payments or refinement under different thresholds for the cost differentials and concentration measures.

In Section 2, we describe how receipt of complex care may have a differential impact on the costs of care for patients allocated to the same HRG and the implications that this might have on hospital profitability. This motivates our empirical approach to estimating cost differentials and in analysing the concentrations of complex care among hospitals and HRGs. We analyse patient‐level data for the 2013/14 financial year and describe these data in Section 3. Section 4 focuses on the results. The policy implications of the results are discussed, limitations acknowledged, and conclusions drawn in Section 5.

## 2 EMPIRICAL FRAMEWORK

Every patient treated in hospital is allocated to a single HRG, which forms the basis by which the hospital is reimbursed for the care provided. An individual patient i=1...I is allocated to a unique HRG h=1...H according to a set of observable patient characteristics, denoted as vector X, including the type of procedure performed (OPCS codes), diagnoses (ICD10 codes), 1ICD10: International Statistical Classification of Diseases and Related Health Problems 10th Revision; OPCS: Office for Population Censuses and Surveys Classification of Surgical Operations and Proceduresthe presence of complications and comorbidities, age, and gender (Grašič, Mason, & Street, 2015).

Patients allocated to the same HRG may differ according to whether they received some form of complex care, identifiable in England according to a set of identification rules known as Prescribed Specialised Services (PSS; NHS England, 2014b). For ease of exposition, we assume initially that there is only one type of complex care and denote $\bar{s}$ to indicate that a patient received complex care and $\underline{s}$ to denote that they did not, such that $S\in (\underline{s},\bar{s})$.

Ceteris parabis for patients allocated to any representative HRG, the cost of hospital care c_ih_ is likely to be higher for patients who required complex care than for those that did not, such that $c_{ih}({\underline{s}}_{i},X_{ih})<c_{ih}({\bar{s}}_{i},X_{ih})$. We anticipate that receipt of complex care will have a proportionate effect β_h_ on costs such that $\beta_{h}c_{ih}({\underline{s}}_{i},X_{ih})=c_{ih}({\bar{s}}_{i},X_{ih})$. The national average cost of an HRG c_h_ will therefore depend on the proportion of patients nationally ρ who receive complex care, such that

(1) $$c_{h}=\rho \times c_{h}(\bar{s},X_{h})+(1-\rho \times c_{h}(\underline{s},X_{h}).$$

In many prospective payment systems, the price p_h_ paid for each patient allocated to a particular HRG is based on average costs (Schreyögg, Stargardt, Tiemann, & Busse, 2006). Thus expected profit (i.e., excess revenue) per patient amounts to π_h_=p_h_−c_h_=0. But expected profit will differ from hospital to hospital, if the proportion of complex care patients that the hospital treats ρ_k_ differs from the national average ρ. So if ρ_k_≠ρ then c_hk_≠c_h_, with the average profit per patient for hospital k=1⋯K given by

(2) $$\pi_{hk}=c_{h}(\underline{s},X_{h})[\rho (\beta_{h}-1)+\rho_{k}(1-\beta_{h})].$$

Profit is negative and increasing in magnitude the larger ρ_k_ relative to ρ and the larger β_h_ relative to 1. Thus, if providing complex care does increase costs, then a policy designed to concentrate complex care activity among fewer hospitals risks being financially punitive to those hospitals providing complex care. Our empirical strategy is, therefore, to estimate the proportionate additional cost β_h_ associated with provision of complex care service and to assess the proportion of complex care activity ρ_k_ undertaken by each hospital.

To do this, we use the reference cost data, a collation of HRG costs reported in a standardised format by official mandate by all English hospitals to the Department of Health (Department of Health, 2012). As in most countries with prospective payment systems (Schreyögg et al., 2006), for each HRG, the cost information comprises two elements: a base cost $c_{h}^{b}$ for those patients with a typical length of stay (LoS) for their HRG and an excess per diem cost $c_{h}^{e}$ for each extra day spent in hospital by patients with an exceptionally long LoS for their HRG (Health and Social Care Information Centre, 2015). Thus, the full cost of hospital treatment for each patient i allocated to HRG h and treated in hospital k takes the form:

(3) $$c_{ihk}=c_{ihk}^{b}+c_{ihk}^{e}d_{ihk},$$

where d_ihk_ indicates the additional number of days that the patient stays in hospital above the typical LoS for their HRG. These reported costs are converted into prices in a similar fashion to that adopted in other countries with a prospective payment system, whereby a base price $p_{h}^{b}$ is made for each patient according to the HRG to which they are allocated, and an additional per diem price $p_{h}^{e}$ is paid for each day spent in hospital beyond the LoS typical to the HRG (Schreyögg et al., 2006). In keeping with these payment arrangements, we investigate whether the provision of complex care influences variation in either base costs or additional per diem costs in separate regression models, denoting these costs as $c_{ihk}^{*}\in (c_{ihk}^{b},c_{ihk}^{e})$.

The empirical challenge in estimating these costs is that patients are admitted to hospital for many different types of treatment hence their categorisation to specific HRGs. One way to account for this diversity is to introduce a dummy variable for each HRG, but because there are so many, categories 2In 2013/14 the HRG system comprised 2100 HRGs.estimation is cumbersome. The alternative is to standardise each patient's cost by the mean cost of all patients allocated to the same HRG (Daidone & Street, 2011a). Thus the dependent variable is defined as

(4) $$y_{ik}^{*}=\frac{c_{ihk}^{*}}{Z_{k}c_{h}^{*}},$$

where $c_{ihk}^{*}$ is the cost of patient i in HRG h in hospital k, and $c_{h}^{*}$ is the national mean cost for patients allocated to HRG h. In the analyses that follow and in keeping with how prices in England and elsewhere are calculated, costs are also purged of geographical variation in wages and in the cost of land and buildings (Mason, Street, Miraldo, & Siciliani, 2009; Zuckerman, Welch, & Pope, 1990). This hospital‐specific adjustment is denoted Z_k_. 3The adjustment is made using the English Department of Health's Market Forces Factor (MFF). https://www.gov.uk/government/uploads/system/uploads/attachment_data/file/214906/PbR-and-the-MFF-in-2013-14.pdf.


To estimate the cost differential associated with receipt of complex care, we regress $y_{ik}^{*}$ against a full set (n=1⋯N) of complex care markers (S_n_) indicating the type of complex care received (if any). 4It is possible for a patient to receive more than one type of complex care service. Receipt of multiple services may lead to correlation problems. However, the number of patients with multiple complex care services markers is very low and Variance Inflation Factors confirm that multi‐collinearity is not a problem.So, for any individual i, S_ni_=1 if the patient received complex care of type n, and 0 otherwise. The random effects model, recognising that patients are clustered within hospitals, takes the form:

(5) $$y_{ik}^{*}=\alpha +\overset{N}{\underset{n=1}{\sum }}\beta_{n}S_{nik}+u_{k}+\epsilon_{ik},$$

where β_n_ are the parameters to be estimated. Costs will be related also to hospital efficiency u_k_ (Laudicella, Olsen & Street, 2010; Street, Kobel, Thuilliez, & Renaud, 2012), which we assume acts like a scaling factor, increasing or decreasing the cost of care for a particular patient, and which is not subject to reimbursement. Costs will also vary from one patient to another for reasons that cannot be observed. These reasons are captured by ϵ, a random error term assumed to have a normal distribution, with E[ϵ_ik_]=0.

If the ${\hat{\beta }}_{n}$ parameters are not significant explanators of variation in cost, they do not need to be taken into account in payment design. But if positive and significant, a patient with complex care marker n has higher costs than do other patients allocated to the same HRG. By defining the dependent variable as a ratio, we can calculate the percentage cost differential associated with receipt of complex care (Daidone & Street, 2013). In order to derive the percentage increase in costs associated with receipt of complex care, g_n_, we compute the marginal mean for both complex care and noncomplex care services (Daidone & Street, 2013):

(6) $$g_{n}=\frac{E({\;y}_{i}|S_{n}=\bar{s},S)-E({\;y}_{i}|S_{n}=\underline{s},S)}{E({\;y}_{i}|S_{n}=\underline{s},S)}\times 100.$$

Even if patients receiving complex care treatment have higher costs, if the distribution of complex care across hospitals is random, hospitals will not be financially disadvantaged in a systematic fashion. But hospitals treating more complex care patients are at risk of receiving insufficient revenue to cover their costs. We assess the concentration of complex care patients by means of a Gini coefficient, G_n_, for each particular type of complex care n, calculated according to the formula:

(7) $$G_{n}=\frac{K+1}{K-1}-2\times \frac{\sum_{k=1}^{K}\tau_{nk}Q_{nk}}{K(K-1)\rho_{n}},$$

where K is the number of hospitals, and ρ_n_ is the mean proportion of complex care patients of type n across all hospitals. The Gini coefficient ranges from 0 (no concentration) to 1 (complex care is concentrated in a single hospital). We rank all hospitals according to their number of complex care patients Q_nk_, with the hospital performing the greatest amount ranked first. τ_k_ is the rank of hospital k with Q_nk_ patients receiving complex care of type n.

If the estimated coefficients ${\hat{\beta }}_{n}$ are significant and patients are concentrated in particular hospitals, the payment system needs to be revised in some way to avoid punitive financial consequences. There are two options: to refine the underlying HRGs to account for the provision of complex care or to apply a compensatory top‐up payment to either or both the base and excess per diem prices. The choice will depend on the extent to which patients receiving complex care are concentrated within existing HRGs. If concentrated in a small number of HRGs, refinement of these HRGs might be feasible. But if these patients are spread across multiple HRGs, subdividing each one will generate many HRGs, giving rise to two risks. First, each group will contain too few patients for valid statistical comparisons, this being a key condition specified by the architects of the original DRG system (Fetter, Shin, Freeman, Averill, & Thompson, 1980). Second, the fewer hospitals treating patients requiring complex care, the greater the risk that the payment system reduces to cost‐based reimbursement. To guard against these risks, when patients receiving complex care are spread across multiple HRGs, a top‐up payment would be preferred.

To assess the concentration of complex care services among different HRGs, we could also construct Gini coefficients. However, because of the large number of HRGs (h=2100), the Gini coefficient will always be very close to 1. Instead, we calculate concentration ratios analogous to the Four‐Firm measures used to measure industry structure (Siegfried, 1975).

The concentration ratio is the percentage of total complex care activity of type n allocated to the four HRGs that account for the largest amount of this type of complex care and is calculated as

(8) $$CR_{n}^{4}=\overset{4}{\underset{h=1}{\sum }}{\bar{s}}_{nh},$$

where ${\bar{s}}_{nh}$ is the share of activity provided in the h‐th largest HRG by volume of complex care activity of type n.

## 3 DATA

In order to assess the costs associated with hospitalised patients receiving complex care services, we analyse data from the patient level Hospital Episode Statistics (HES) for 2013/14 matched to reference cost (RC) data reported by all English hospitals. These costs are purged of exogenous cost factors. The HES contains details about every patient treated in the English NHS during the financial year. Each patient observation constitutes their time in hospital from admission to discharge.

We identify what type of complex care service, if any, each patient received by applying the set of PSS identification rules (NHS England, 2014b). Information in each patient's first diagnostic and all procedural fields is examined to ascertain whether the ICD10 or OPCS codes designated in the PSS are present in their medical record. In general, these codes differ from those used for HRG assignment. The PSS manual and accompanying spreadsheet 5
http://www.hscic.gov.uk/media/11878/PS-201314-Identification-Code-Sets/xls/PS_2013_14_Prescribed_Services_Identification_Code_Sets_v1.1.xlsx.
set out identification rules for 143 groups of complex care services, of which 69 relate to services for patients admitted to hospital (NHS England, 2014b). The PSS 2013‐14 identification tool is available online. 6
http://www.hscic.gov.uk/casemix/prescribedspecialisedservices



We match each patient's HES record to the RC reported by their hospital in order to establish the base, $c_{ihk}^{b}$, and excess per diem, $c_{ihk}^{e}$, costs of their hospital care. Only 4.1% of patients have excess per diem costs. 7Almost 1.4 m patients also have unbundled costs, these being associated with high cost services or procedures. These are paid for separately and, hence, are omitted from the analyses.Matching of costs to patients is done through a combination of the hospital code, point of delivery (e.g., day case, elective, and nonelective), specialty (e.g., 300: General Surgery) and HRG code. 8
https://www.gov.uk/government/publications/reference-costs-guidance-for-2011-12



The matched HES and RC data yielded 12,474,184 patients, of which 792,974 received a complex care service of one form or another. However, 25,362 (3.2%) of patients that received complex care were allocated to HRGs in which all patients received complex care because the PSS rules coincided with those used to construct these HRGs. This was true, for instance, of almost everyone having bone marrow transplantation, cochlear implants and bone anchoring hearing aids. Given that all those allocated to these HRGs received complex care, there is no cost differential to estimate. Hence, patients allocated to HRGs in which everyone received complex care are dropped from the analysis. This leaves an analytical sample of 12,403,818 patients, of which 766,204 received complex care.

## 4 RESULTS

In Table 1, we report the number of patients in the analysis for each complex care marker 9Results are not shown if fewer than 300 patients nationally received this type of complex care, of which there were 14 types; results for these complex care markers are available from the authors on request. and the results of applying Equation 6 to the estimates derived from Equation 5, highlighting significant cost differentials (p<.0001). This shows, for example, that 41,389 patients (N base cost) received chemotherapy and that the base cost of their hospital care was 4% higher than otherwise similar patients allocated to the same HRG. Of these patients, 202 (N per diem) stayed in hospital longer than typical for their HRG, and their excess per diem cost was 15% higher than that for otherwise similar patients allocated to the same HRG. Many hospitals provide chemotherapy, the Gini coefficient being 0.66, and patients receiving this service are allocated to only a handful of HRGs, the four main HRGs (CR4) accounting for 98% of this complex care activity.

**Table 1:** Patient numbers, regression results, and concentration measures^a^

	N base cost	Base cost differential (%)	N per diem	Per diem differential (%)	Gini	CR4
Chemotherapy	41,389	**4**	202	**15**	0.66	0.98
PET‐CT^b^	497	**132**	129	−2	0.95	0.11
Radiotherapy	10,536	**37**	822	4	0.97	0.86
Stereotactic radiosurgery	1,197	**−112**	9	−16	0.99	0.84
Teenage and young adults cancer	5,864	**28**	187	2	0.82	0.56
Rare cancers (adult)	30,019	**17**	1812	−3	0.72	0.42
Haemophilia	3,673	**23**	115	**29**	0.89	0.74
Women—complex minimal access gynaecology surgery	1,961	**−8**	13	2	0.77	0.59
Women—maternal medicine	41,612	**9**	1743	0	0.66	0.75
Spinal—spinal surgery	8,291	**−14**	667	−1	0.84	0.49
Neurosciences—neurology	121,574	**10**	6218	**14**	0.86	0.52
Neurosciences—neurosurgery	61,308	**41**	4441	**3**	0.92	0.24
Burns care	2,009	**73**	256	**23**	0.97	0.88
Cystic fibrosis	422	−9	8	−14	0.87	0.76
Renal services—access for dialysis	12,255	**20**	557	4	0.88	0.43
Renal services—renal Transplantation	8,793	**−17**	258	11	0.88	0.8
Cardiac—cardiac electrophysiology	6,028	−3	462	**−10**	0.88	0.73
Cardiac—inherited heart disorders	4,162	**16**	262	0	0.69	0.43
Cardiac—cardiac surgery	27,354	**22**	1432	**−7**	0.9	0.26
Cardiac—PPCI and Structural Heart Disease^c^	27,337	**13**	2086	−3	0.79	0.8
Cardiac—pulmonary hypertension	906	**12**	12	−3	0.93	0.7
Cardiac—cardiovascular magnetic resonance	1,483	**60**	240	10	0.93	0.27
Cardiac—other	19,567	2	814	3	0.68	0.47
Adult congenital heart disease	3,507	**−14**	126	−9	0.85	0.65
Cleft lip palate	2,275	**−7**	59	−2	0.95	0.7
Immunology	9,140	**−9**	27	25	0.92	0.95
Allergy	2,292	**−22**	0	0%	0.97	0.77
Hepatology and pancreatic	2,847	**8**	188	−2	0.84	0.6
Children—cancer	20,510	**11**	392	−1	0.9	0.7
Children—cardiac	7,283	**18**	501	**18**	0.85	0.42
Children—endocrinology	4,270	**−8**	20	**65**	0.96	0.85
Children—gastroenterology	54,635	**4**	2133	−1	0.72	0.33
Children—haematology	1,754	**8**	43	−13	0.96	0.73
Children—neurosciences	11,010	**24**	528	−1	0.96	0.24
Children—ophthalmology	6,467	**30**	100	−2	0.8	0.31
Children—renal	7,205	**−9**	229	11	0.97	0.64
Children—respiratory	9,139	**30**	424	**15**	0.95	0.46
Children—rheumatology	7,376	−1	204	10	0.84	0.51
Children—surgery	59,543	**15**	5329	**5**	0.79	0.38
Respiratory—complex thoracic surgery	27,562	**35**	1200	4	0.9	0.32
Respiratory—management of central airway obstruction	2,206	**47**	127	**31**	0.84	0.38
Respiratory—interstitial lung disease	9,330	−2	468	−2	0.65	0.72
Respiratory—other	16,373	**13**	1609	0	0.6	0.2
Vascular Services	5,577	**7**	506	6	0.75	0.58
Colorectal—incontinence	1,270	**−42**	3	−43	0.95	0.77
Colorectal—transanal endoscopic microsurgery	497	**55**	2	−32	0.91	0.86
Orthopaedic surgery	1,628	**20**	196	−4	0.79	0.38
Morbid obesity surgery Ophthalmology	23,022	**−2**	729	**−10**	0.78	0.25
Haemoglobinopathy—sickle cell	10,813	**10**	601	7	0.95	0.73
Haemoglobinopathy—thalassaemia	8,121	0	7	34	0.9	1
Highly specialised	8,014	**29**	385	**59**	0.99	0.16
Total specialised patients	766,204		39,007			
Total patients	12,403,818					

a Includes complex care services markers for which the sample is more than 300 patients.

b Positron emission tomography‐computed tomography.

c Primary Percutaneous coronary intervention.

As shown in the column reporting the base cost differential, many, but not all, of the complex care markers are positive and significant when considering base costs. The cost differential between those that do and do not receive complex care is more than 10% for 26 markers; for 12 of these, the difference is more than 25%; and for 4 it is more than 50%, these being positron emission tomography computed tomography (132% higher), burns care (73%), cardiovascular magnetic resonance (60%), and colorectal transanal endoscopic microsurgery (55%).

The excess per diem cost differential between those that do and do not receive complex care is more than 10% for nine markers; for four of these, the difference is more than 25%; and for 2, it is more than 50%, these being children's endocrinology (65%) and highly specialised care (59%).

The Gini coefficients show that most complex care services seem to be concentrated among relatively few hospitals with a mean value of Gini = 0.88. The minimum Gini coefficient is 0.60 (for respiratory—other) and the maximum is 0.99 (highly specialised). Given that complex care activity is generally highly concentrated within particular hospitals, it is clear that failing to compensate for the additional costs of complex care activity would not affect all hospitals equally.

For a handful of complex care markers, the additional cost estimates are negative, suggesting that patients receiving these forms of complex care are less costly than other patients allocated to the same HRG. This is particularly notable for stereotactic radiosurgery (−112%), colorectal incontinence (−42%), and renal transplantation (−17%). Care is highly concentrated for these services, the Gini coefficients being 0.99, 0.95, and 0.88 respectively. Those hospitals in which care these types of complex care service are concentrated might benefit from economies of scale, in which case they would have lower differential costs. There is previous research suggesting that hospitals specialising in fewer categories of medical services have lower costs (Farsi & Filippini, 2008).

In Table 2, we report our estimates of the total costs at national level for those complex care markers for which estimates of the additional costs were positive and significant. For the NHS as a whole, the additional costs associated with provision of complex care amount to £588 m representing 3.5% of the funding allocated through the prospective payment system (Monitor, 2015). The national financial impact exceeds £70 m for three types of complex care these being neurosurgery (£157 m), cardiac surgery (£79 m) and complex thoracic surgery (£75 m).

**Table 2:** Financial impact and refinement recommendations^a^

			Total		Base cost		
	Gini	CR4	value (£000)	N nationally	differential (%)	Impact (£000)	Recommendation
Chemotherapy	0.66	0.98	£34,112	782,697	**4**	£1,327	
PET‐CT^b^	0.95	0.11	£4,586	1,290	**132**	£6,040	Top‐up
Radiotherapy	0.97	0.86	£54,589	101,472	**37**	£20,430	Split
Teenage and young adults cancer	0.82	0.56	£12,805	13,456	**28**	£3,579	Top‐up
Rare cancers (adult)	0.72	0.42	£132,350	64,478	**17**	£22,775	Top‐up
Haemophilia	0.89	0.74	£5,444	5,585	**23**	£1,229	Top‐up
Women—maternal medicine	0.66	0.75	£44,930	45,142	**9**	£3,947	
Neurosciences—neurology	0.86	0.52	£208,868	144,318	**10**	£21,308	Top‐up
Neurosciences—neurosurgery	0.92	0.24	£383,066	79,986	**41**	£157,190	Top‐up
Burns care	0.97	0.88	£20,910	6,797	**73**	£15,168	Split
Renal services—access for dialysis	0.88	0.43	£49,893	17,203	**20**	£10,084	Top‐up
Cardiac—inherited heart disorders	0.69	0.43	£30,249	6,260	**16**	£4,767	Top‐up
Cardiac—cardiac surgery	0.9	0.26	£365,229	43,488	**22**	£79,265	Top‐up
Cardiac—PPCI^c^ and structural heart disease	0.79	0.8	£189,369	50,482	**13**	£25,527	Split
Cardiac—pulmonary hypertension	0.93	0.7	£3,342	1,153	**12**	£391	Top‐up
Cardiac—cardiovascular magnetic resonance	0.93	0.27	£22,560	6,123	**60**	£13,612	Top‐up
Hepatology and pancreatic	0.84	0.6	£32,480	4,178	**8**	£2,455	
Children—cancer	0.9	0.7	£65,046	55,693	**11**	£7,210	Top‐up
Children—cardiac	0.85	0.42	£47,916	18,169	**18**	£8,660	Top‐up
Children—gastroenterology	0.72	0.33	£136,175	82,217	**4**	£5,139	
Children—haematology	0.96	0.73	£3,507	2,493	**8**	£290	
Children—neurosciences	0.96	0.24	£40,557	16,824	**24**	£9,576	Top‐up
Children—ophthalmology	0.8	0.31	£8,983	8,676	**30**	£2,727	Top‐up
Children—respiratory	0.95	0.46	£14,490	11,825	**30**	£4,293	Top‐up
Children—surgery	0.79	0.38	£312,937	137,885	**15**	£45,840	Top‐up
Respiratory—complex thoracic surgery	0.9	0.32	£214,554	37,283	**35**	£74,736	Top‐up
Respiratory—management of central airway obstruction	0.84	0.38	£11,300	3,428	**47**	£5,336	Top‐up
Respiratory—other	0.6	0.2	£133,625	27,326	**13**	£17,791	Top‐up
Vascular services	0.75	0.58	£76,998	7,742	**7**	£5,457	
Colorectal—transanal endoscopic microsurgery	0.91	0.86	£1,437	543	**55**	£789	Split
Orthopaedic Surgery	0.79	0.38	£9,747	1,942	**20**	£1,992	Top‐up
Haemoglobinopathy—sickle cell	0.95	0.73	£20,717	19,947	**10**	£2,025	
Highly specialised	0.99	0.16	£22,453	12,067	**29**	£6,569	Top‐up
Total			£2,715,227			£587,524	

a Includes complex care markers for which the sample is more than 300 patients and where the cost differential is positive and significant.

b Positron emission tomography‐computed tomography.

c Primary Percutaneous coronary intervention.

**Figure 1:**
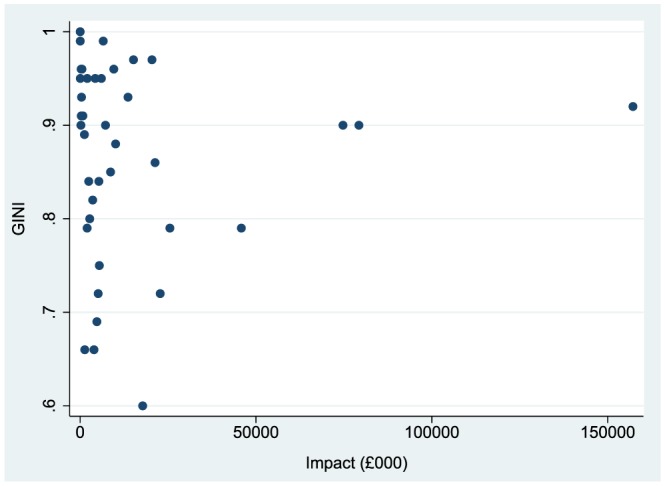
Gini coefficient and financial impact of of Prescribed Specialised Services markers

In Figure 1, we plot each of the complex care markers in terms of its national financial impact and the Gini coefficient measuring hospital concentration. The above‐mentioned three complex care markers stand out in the upper right corner because these types of complex care are conducted in few hospitals (Gini >0.8) and have a high national financial impact. Failure to account for the additional costs associated with their complex care would therefore have a substantial punitive effect on those few hospitals that provide these complex care services.

In view of these findings, there is a strong case for compensating complex care activity on the basis of the observed cost differentials, and the impact of these higher costs may have on individual hospitals. We explore two options by which these extra costs could be compensated. The current convention is to apply a top‐up to existing HRGs (Monitor & NHS England, 2013), but it is also worth considering whether it would be preferable to further refine the underlying HRGs to which patients receiving complex care are allocated (Hafsteinsdottir & Siciliani, 2010). To assess these alternatives, we calculate the CR4 ratio, measuring the proportion of complex care services that are in the most common four HRGs. The CR4 ratios are reported in the final column of Table 1.

The choice between whether to adopt top‐up payments or refine HRGs requires making decisions about what size of cost differential is deemed material and how concentrated services need to be among hospitals and HRGs before a split is considered. For example, for illustrative purposes, suppose that the threshold for the cost differential is 10%, and for both the Gini and CR4, it is >0.8. 26 of the 69 complex care services have a cost differential in excess of 10%. At the 0.8 concentration thresholds, top‐up payments to the HRG price would be considered for 23 of the 26 types of complex care. This payment would be made in proportion to the estimated cost differential associated with the type of complex care in question with offsetting reductions in the base price for other patients allocated to the same HRG. HRG splits would be considered for three of the 26 types of complex care, namely, radiotherapy (Gini = 0.97 & CR4 = 0.86), burns care (Gini = 0.97 & CR4 = 0.88), and colorectal transanal endoscopic microsurgery (Gini = 0.91 & CR4 = 0.86). With patients receiving these particular complex care service being allocated to just a few HRGs, these might be subdivided to distinguish patients that receive complex care from those that do not.

**Figure 2:**
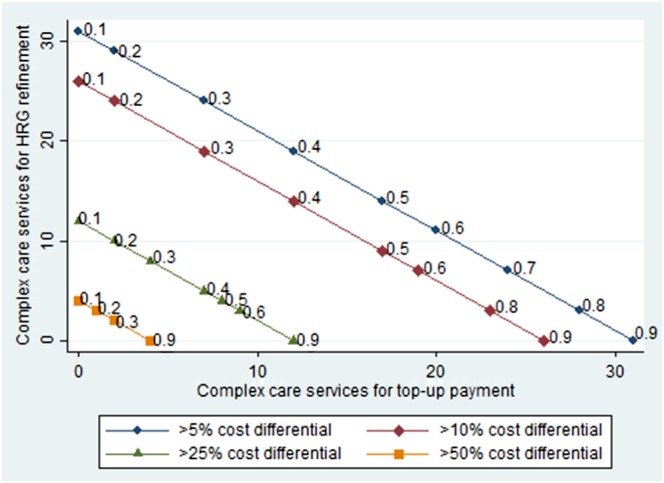
Complex care services considered for Healthcare Resource Groups refinement or top‐up payments according to choices about the size of the cost differential and concentration measures

Figure 2 illustrates how the number of complex care services that are candidates for HRG refinement or top‐up payments varies according to decisions about the size of the cost differential and concentration measures. The graph shows four lines indicating the number of complex care services with cost differentials in excess of 50%, 25%, 10%, and 5%. For each line, the number of complex care services considered for either refinement or top‐up payments can be identified according to thresholds chosen for the two concentration measures. Thresholds (rounded to a single decimal place) are shown along each line if these indicate a change in the number of complex care services considered for either HRG refinement or top‐up payments.

## 5 CONCLUSIONS

The policy for the English NHS of concentrating complex care services in particular providers is designed to improve outcomes for people with relatively rare and complex conditions. But the payment system needs to be aligned with this policy ambition so that hospitals that provide complex care are not penalised financially for doing so.

There is no universally agreed definition of what constitutes complex care hospital care, but in England attempts have been made to define complex care according to the presence of specific diagnoses and procedures in each patient's medical record. We have applied these complex care definitions to determine whether the receipt of complex care is associated with higher costs relative to patients allocated to the same HRG who did not receive complex care. To do this, we estimate random effects models using patient‐level activity and cost data for all patients admitted to English hospitals during the 2013/14 financial year. Compared to otherwise equivalent patients allocated to the same HRG, costs were more than 10% higher for patients receiving 26 (out of 69) types of complex care delivered in hospitals.

The existence of cost differentials is a necessary but not sufficient condition for refining the payment system. In addition, we consider materiality, in terms of both the national financial impact and the number of hospitals that will be affected. These dual aspects are important because, even though there may not be many patients nationally receiving a particular type of complex care service, if these patients are concentrated in few hospitals, payments may have a material impact on their income and ability to provide the service. For those complex care markers for which the estimated cost differential is deemed to have a material impact, we explore two ways in which payment policy might be refined.

First, HRGs might be refined so that they better separate higher cost patients that receive complex care from those that do not (Hafsteinsdottir & Siciliani, 2010). If perfect separation can be achieved, the prices for HRGs in which everybody receives complex care will adequately reflect the expected costs associated with complex care. This strategy is most easily adopted for those types of complex care where patients are concentrated in a limited number of HRGs, evaluated here by calculating the proportion of complex care activity concentrated among the four largest HRGs to which patients are assigned. If, by way of illustration, the cost differential exceeds 10%, and the concentration measures exceed 0.8, HRG splits would be considered for three types of complex care and top‐up payments for 23 types. If these thresholds were gradually lowered, yet more HRGs would be constructed to account for progressively more of these types of complex care services. But the risk of constructing ever more refined HRGs is that the likelihood of one hospital treating all patients allocated to a specific HRG increases, the consequence being that the HRG price collapses to cost‐based reimbursement.

Subdividing HRGs cannot be adopted easily for those types of complex care where patients are distributed across many HRGs. In these cases, prices rather than HRGs might be refined, with top‐up payments made to reflect the additional costs associated with receipt of complex care. Top‐up payments can be made to either or both the base and excess *per diem* prices, according to where the cost differentials are observed.

There are opportunities to improve on this work, notably by improving the cost data. There is a large U.S. literature analysing hospital costs that relies on charge data (Frakt, 2011). RC data are analogous to the charge data reported to the Healthcare Cost Report Information System. 10
http://www.cms.gov/Medicare/Medicare-Fee-for-Service-Payment/AcuteInpatientPPS/FY2015-IPPS-Final-Rule-Home-Page-Items/FY2015-Final-Rule-Data-Files.html
Neither U.S. charge data nor English RC data capture precisely the costs of care for each individual patient (Dunham‐Taylor & Pinczuk, 2006). The limitation of using RC data is that patients that share the same characteristics, such as hospital, specialty, point of delivery, HRG, and LoS, will be assigned the same RC by their hospital. For the analysis, this means that the RC data exhibit less variation than occurs in reality. If some of this unobserved variation is related to the receipt of complex care, then the estimates of the cost differentials will be biased, most probably in a downward direction. Recognising this limitation, we also analysed variation in LoS, but found that cost differentials are more likely to be observed than LoS differentials (Bojke, Grašič, & Street, 2015).

Patient‐level information and costing systems (PLICS) could alleviate the drawback of using RC data, as patient‐level costs ought to take account of more specific drivers of resource use than do the RC. But PLICS will not resolve the problem entirely because judgments still have to be made about how to apportion shared costs among individual patients (Jackson, 2001). Also, on a practical level, PLICS will not be available in England for all patients in the near future. PLICS reporting is currently not mandatory, and for the year of data, we consider (2013/14) only 53% of hospitals were reporting patient level costs 11
https://www.gov.uk/government/uploads/system/uploads/attachment_data/file/380322/01_Final_2013-14_Reference_Costs_publication_v2.pdf
; and these were not representative of the overall population of hospitals. 12
http://www.ehi.co.uk/Features/item.cfm?&docId=404



As patient‐level cost data become available, payment arrangements can be progressively refined. Refinements might involve construction of more resource homogenous HRGs and better calculation of the base and excess *per diem* prices associated with each HRG. In the meantime, our analyses indicate for which types of complex care services refinements are required to current HRG prices so that policy ambitions to further the concentration of complex care services are not thwarted by an inadequate payment system. The results have been used to inform hospital payment arrangements in England for 2016/17 (Monitor, 2015).

## ETHICAL APPROVAL STATEMENT

This project used only secondary administrative data. All patient‐level data supplied to us by the Health and Social Care Information Centre were psuedonymised, and no personal information was included. All results are reported at aggregated levels and complied with reporting requirements on disclosure of potentially patient identifiable data. As the projects do not involve patients directly and patients are not potentially identifiable from the data, ethical approval was not required for this project.

## Supporting information

Supporting info item
