# Supplementary material for: How should hospital reimbursement be refined to support concentration of complex care services?
Source: Health Econ. 2017 May 19;27(1):e26–38. doi: 10.1002/hec.3525 (PMC5836989; doi:10.1002/hec.3525)
Supplement: Supplementary file 1 — Supporting info item [file HEC-27-e26-s001.pdf]

Table 1: Regression results: all markers

	Col(1)	Col(2)	Col(3)	Col(4)	Col(5)	Col(6)
Chemotherapy	41,389	**4%**	202	**15%**	0.66	0.98
PET-CT*	497	**132%**	129	-2%	0.95	0.11
Radiotherapy	10,536	**37%**	822	4%	0.97	0.86
Stereotactic Radiosurgery	1,197	**-112%**	9	-16%	0.99	0.84
Teenage and Young Adults Cancer	5,864	**28%**	187	2%	0.82	0.56
Rare Cancers (Adult)	30,019	**17%**	1812	-3%	0.72	0.42
Bone Marrow Transplantation	3	**373%**	98	-6%	0.9	0.86
Haemophilia	3,673	**23%**	115	**29%**	0.89	0.74
Women - Complex Minimal Access Gynaecology Surgery	1,961	**-8%**	13	2%	0.77	0.59
Women - Fetal Medicine	125	**123%**	8	21%	0.96	0.84
Women - Complex Urinary and Faecal Incontinence & Genital Prolapse	35	-13%	4	-17%	0.93	0.77
Women - Maternal Medicine	41,612	**9%**	1743	0%	0.66	0.75
Spinal - Spinal Surgery	8,291	**-14%**	667	-1%	0.84	0.49
Neurosciences - Neurology	121,574	**10%**	6218	**14%**	0.86	0.52
Neurosciences - Neurophysiology	232	**-21%**	5	-41%	0.99	0.89
Neurosciences - Neuroradiology	13	**160%**	0	0%	0.99	0.93
Neurosciences - Neurosurgery	61,308	**41%**	4441	**3%**	0.92	0.24
Burns Care	2,009	**73%**	256	**23%**	0.97	0.88
Cystic fibrosis	422	-9%	8	-14%	0.87	0.76
Renal Services - Access for dialysis	12,255	**20%**	557	4%	0.88	0.43
Renal Services - Renal Transplantation	8,793	**-17%**	258	11%	0.88	0.8
Cardiac - Cardiac electrophysiology	6,028	-3%	462	**-10%**	0.88	0.73
Cardiac - Inherited heart disorders	4,162	**16%**	262	0%	0.69	0.43
Cardiac - Cardiac surgery	27,354	**22%**	1432	**-7%**	0.9	0.26
Cardiac - PPCI** and Structural Heart Disease	27,337	**13%**	2086	-3%	0.79	0.8
Cardiac - Pulmonary hypertension	906	**12%**	12	-3%	0.93	0.7
Cardiac - Cardiovascular magnetic resonance	1,483	**60%**	240	10%	0.93	0.27
Cardiac - Other	19,567	2%	814	3%	0.68	0.47
Adult Congenital Heart Disease	3,507	**-14%**	126	-9%	0.85	0.65
Cleft Lip Palate	2,275	**-7%**	59	-2%	0.95	0.7
Immunology	9,140	**-9%**	27	25%	0.92	0.95
Allergy	2,292	**-22%**	0	0%	0.97	0.77
Infectious Diseases Adult	241	**-22%**	11	11%	0.97	0.78
Infectious Diseases Paeds	111	18%	9	-10%	0.97	0.5
Hepatology & Pancreatic	2,847	**8%**	188	-2%	0.84	0.6
Mental Health - Gender Dysphoria	175	5%	0	0%	1	1
Children - Cancer	20,510	**11%**	392	-1%	0.9	0.7
Children - Cardiac	7,283	**18%**	501	**18%**	0.85	0.42
Children - Endocrinology	4,270	**-8%**	20	**65%**	0.96	0.85
Children - Gastroenterology	54,635	**4%**	2133	-1%	0.72	0.33
Children - Haematology	1,754	**8%**	43	-13%	0.96	0.73
Children - Neurosciences	11,010	**24%**	528	-1%	0.96	0.24
Children - Ophthalmology	6,467	**30%**	100	-2%	0.8	0.31
Children - Renal	7,205	**-9%**	229	11%	0.97	0.64
Children - Respiratory	9,139	**30%**	424	**15%**	0.95	0.46
Children - Rheumatology	7,376	-1%	204	10%	0.84	0.51
Children - Surgery	59,543	**15%**	5329	**5%**	0.79	0.38
Childrens services - Paediatric Pain Management	18	**82%**	0	0%	1	0.71
Hyperbaric Oxygen Treatment	8	24%	1	-9%	1	1
Respiratory - Pulmonary vascular services	23	-37%	1	-4%	0.96	0.79
Respiratory - Complex thoracic surgery	27,562	**35%**	1200	4%	0.9	0.32
Respiratory - Management of central airway obstruction	2,206	**47%**	127	**31%**	0.84	0.38
Respiratory - Interstitial lung disease	9,330	-2%	468	-2%	0.65	0.72
Respiratory - Other	16,373	**13%**	1609	0%	0.6	0.2
Vascular Services	5,577	**7%**	506	6%	0.75	0.58
Ears - Cochlear Implants	49	**68%**	106	**-27%**	0.95	0.99
Ears - Bone anchored hearing aids	79	4%	7	5%	0.87	1
Ears - Middle Ear Implants	98	12%	3	-20%	0.89	0.94
Colorectal - Incontinence	1,270	**-42%**	3	-43%	0.95	0.77
Colorectal - Complex Inflammatory Bowel disease	75	-8%	3	-12%	0.86	0.69
Colorectal - Transanal Endoscopic Microsurgery	497	**55%**	2	-32%	0.91	0.86
Orthopaedic Surgery	1,628	**20%**	196	-4%	0.79	0.38
Orthopaedic Surgery - revisions	123	**32%**	21	7%	0.91	0.54
Morbid Obesity Surgery	4,301	0%	126	0%	0.89	0.97
Ophthalmology	23,022	**-2%**	729	**-10%**	0.78	0.25
Haemoglobinopathy - Sickle Cell	10,813	**10%**	601	7%	0.95	0.73
Haemoglobinopathy - Thalassaemia	8,121	0%	7	34%	0.9	1
Highly Specialised	8,014	**29%**	385	**59%**	0.99	0.16
Total	766,204		39,007			

*Positron emission tomography-computed tomography

**Primary Percutaneous coronary intervention

Table 2: Financial impact in 2013/14: All markers

	GINI	CR4	**Total value (£000)**	Q_n	g_n	**Impact (£000)**
Chemotherapy	0.66	0.98	£34,112	782,697	**4%**	£1,327
PET-CT*	0.95	0.11	£4,586	1,290	**132%**	£6,040
Radiotherapy	0.97	0.86	£54,589	101,472	**37%**	£20,430
Stereotactic Radiosurgery	0.99	0.84	£12,563	1,628	**-112%**	-£14,026
Teenage and Young Adults Cancer	0.82	0.56	£12,805	13,456	**28%**	£3,579
Rare Cancers (Adult)	0.72	0.42	£132,350	64,478	**17%**	£22,775
Bone Marrow Transplantation	0.9	0.86	£60	3,378	**373%**	£226
Haemophilia	0.89	0.74	£5,444	5,585	**23%**	£1,229
Women - Complex Minimal Access Gynaecology Surgery	0.77	0.59	£6,272	2,244	**-8%**	-£528
Women - Fetal Medicine	0.96	0.84	£424	145	**123%**	£521
Women - Complex Urinary and Faecal Incontinence & Genital Prolapse	0.93	0.77	£133	35	-13%	-£17
Women - Maternal Medicine	0.66	0.75	£44,930	45,142	**9%**	£3,947
Spinal - Spinal Surgery	0.84	0.49	£113,755	11,690	**-14%**	-£15,456
Neurosciences - Neurology	0.86	0.52	£208,868	144,318	**10%**	£21,308
Neurosciences - Neurophysiology	0.99	0.89	£386	237	**-21%**	-£80
Neurosciences - Neuroradiology	0.99	0.93	£25	15	**160%**	£41
Neurosciences - Neurosurgery	0.92	0.24	£383,066	79,986	**41%**	£157,190
Burns Care	0.97	0.88	£20,910	6,797	**73%**	£15,168
Cystic fibrosis	0.87	0.76	£4,295	13,696	-9%	-£367
Renal Services - Access for dialysis	0.88	0.43	£49,893	17,203	**20%**	£10,084
Renal Services - Renal Transplantation	0.88	0.8	£65,100	11,852	**-17%**	-£11,114
Cardiac - Cardiac electrophysiology	0.88	0.73	£34,771	7,235	-3%	-£1,046
Cardiac - Inherited heart disorders	0.69	0.43	£30,249	6,260	**16%**	£4,767
Cardiac - Cardiac surgery	0.9	0.26	£365,229	43,488	**22%**	£79,265
Cardiac - PPCI** and Structural Heart Disease	0.79	0.8	£189,369	50,482	**13%**	£25,527
Cardiac - Pulmonary hypertension	0.93	0.7	£3,342	1,153	**12%**	£391
Cardiac - Cardiovascular magnetic resonance	0.93	0.27	£22,560	6,123	**60%**	£13,612
Cardiac - Other	0.68	0.47	£71,918	25,755	2%	£1,460
Adult Congenital Heart Disease	0.85	0.65	£25,887	6,356	**-14%**	-£3,535
Cleft Lip Palate	0.95	0.7	£10,877	2,930	**-7%**	-£711
Immunology	0.92	0.95	£9,997	14,550	**-9%**	-£943
Allergy	0.97	0.77	£2,128	3,809	**-22%**	-£473
Infectious Diseases Adult	0.97	0.78	£1,303	379	**-22%**	-£292
Infectious Diseases Paeds	0.97	0.5	£445	203	18%	£79
Hepatology & Pancreatic	0.84	0.6	£32,480	4,178	**8%**	£2,455
Mental Health - Gender Dysphoria	1	1	£659	175	5%	£31
Children - Cancer	0.9	0.7	£65,046	55,693	**11%**	£7,210
Children - Cardiac	0.85	0.42	£47,916	18,169	**18%**	£8,660
Children - Endocrinology	0.96	0.85	£3,898	4,548	**-8%**	-£317
Children - Gastroenterology	0.72	0.33	£136,175	82,217	**4%**	£5,139
Children - Haematology	0.96	0.73	£3,507	2,493	**8%**	£290
Children - Neurosciences	0.96	0.24	£40,557	16,824	**24%**	£9,576
Children - Ophthalmology	0.8	0.31	£8,983	8,676	**30%**	£2,727
Children - Renal	0.97	0.64	£14,173	19,547	**-9%**	-£1,277
Children - Respiratory	0.95	0.46	£14,490	11,825	**30%**	£4,293
Children - Rheumatology	0.84	0.51	£33,854	8,911	-1%	-£375
Children - Surgery	0.79	0.38	£312,937	137,885	**15%**	£45,840
Childrens services - Paediatric Pain Management	1	0.71	£24	21	**82%**	£20
Hyperbaric Oxygen Treatment	1	1	£5	11	24%	£1
Respiratory - Pulmonary vascular services	0.96	0.79	£205	33	-37%	-£77
Respiratory - Complex thoracic surgery	0.9	0.32	£214,554	37,283	**35%**	£74,736
Respiratory - Management of central airway obstruction	0.84	0.38	£11,300	3,428	**47%**	£5,336
Respiratory - Interstitial lung disease	0.65	0.72	£26,696	12,007	-2%	-£538
Respiratory - Other	0.6	0.2	£133,625	27,326	**13%**	£17,791
Vascular Services	0.75	0.58	£76,998	7,742	**7%**	£5,457
Ears - Cochlear Implants	0.95	0.99	£87	1,086	**68%**	£59
Ears - Bone anchored hearing aids	0.87	1	£259	1,513	4%	£10
Ears - Middle Ear Implants	0.89	0.94	£142	101	12%	£17
Colorectal - Incontinence	0.95	0.77	£5,206	1,826	**-42%**	-£2,200
Colorectal - Complex Inflammatory Bowel disease	0.86	0.69	£772	91	-8%	-£62
Colorectal - Transanal Endoscopic Microsurgery	0.91	0.86	£1,437	543	**55%**	£789
Orthopaedic Surgery	0.79	0.38	£9,747	1,942	**20%**	£1,992
Orthopaedic Surgery - revisions	0.91	0.54	£998	138	**32%**	£318
Morbid Obesity Surgery	0.89	0.97	£21,148	6,809	0%	£21
Ophthalmology	0.78	0.25	£33,233	24,435	**-2%**	-£696
Haemoglobinopathy - Sickle Cell	0.95	0.73	£20,717	19,947	**10%**	£2,025
Haemoglobinopathy - Thalassaemia	0.9	1	£5,638	9,658	0%	£12
Highly Specialised	0.99	0.16	£22,453	12,067	**29%**	£6,569
Total			£2,715,227		£587,524	

*Positron emission tomography-computed tomography

*Primary Percutaneous coronary intervention
